# Time Series Analysis in Modeling of Hepatitis B Incidence

**Published:** 2019-03

**Authors:** Hua ZHANG, Mingdong HUO, Yao TONG, Pei LIU

**Affiliations:** 1. Department of Medical Insurance, School of Public Health, Southeast University, Nanjing, Jiangsu, China; 2. Key Laboratory of Environmental Medicine Engineering, Ministry of Education, School of Public Health, Southeast University, Nanjing, China; 3. Zhongda Hospital Affiliated to Southeast University, Nanjing, Jiangsu, China; 4. Department of Epidemiology and Biostatistics, School of Public Health, Southeast University, Nanjing, Jiangsu, China

## Dear Editor-in-Chief

With up to 240 million people chronically infected with hepatitis B worldwide (1), Hepatitis B is considered to be one of the most serious infectious diseases. “China was a high endemic area of hepatitis B virus infection, around 100 million people (about 10% of the Chinese population) are chronically infected” (2). In the study of infectious diseases, using time series to construct related prediction model has been very common and valuable (3, 4).

In this study, time series analysis was carried out to explore the epidemic trend and improve the prediction accuracy of hepatitis B in Jiangsu Province. Incidence data from 1990 to 2013 came from the Infectious Diseases Information System, SPSS 19.0 (Chicago, IL, USA) was used for statistical analysis. The study will provide basis for policy making of hepatitis B control.

From the following figure and run test, we can see that the sequence was non-stationary. Through the logarithmic conversion and second-order non-seasonal differences, it was made smooth. The white noise test was done by referring to the residual value of the fitting data, and the accuracy of the model was investigated.

**Fig. 1:**
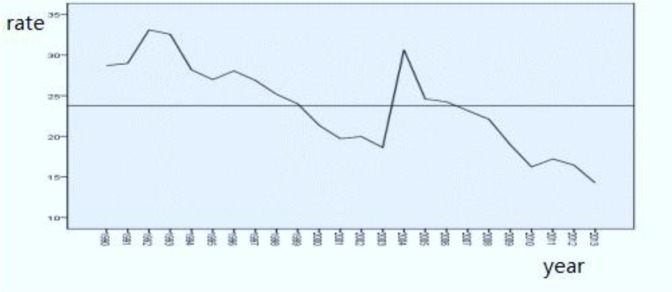
1990–2013 incidence of hepatitis B in Jiangsu Province

**Fig. 2:**
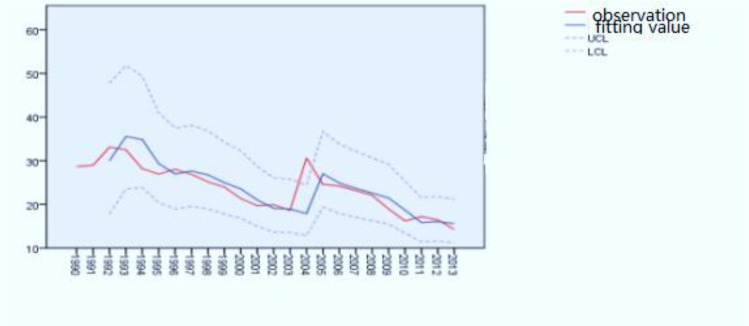
Model fitting trajectories

The incidence of hepatitis B in Jiangsu Province from 1990 to 2013 had a trend of change. The ARIMA [0, 2, 1] model was constructed and fit well. The correlation function was in 95% confidence interval. The dynamic trend trajectories of fitting values of ARIMA [0, 2, 1] model are basically consistent with the trajectories of the actual values (Figs. 1, 2).

Incidence of hepatitis B in Jiangsu has declined in recent years, and prevention measure had positive effect. ARIMA model is applicable in the prediction of hepatitis B and can provide a basis for short-term forecasting. Control strategy should continue to be consolidated in the future.

## References

[1] Sehr MA Joshi KD Fontanesi JM (2017). Markov modeling in hepatitis B screening and linkage to care. Theor Biol Med Model, 14: 11. 28521828 10.1186/s12976-017-0057-6 PMC5437626

[2] Chen G Joan MB Alison AE (2014). Gateway to Care campaign: a public health initiative to reduce the burden of hepatitis B in Haimen City, China. BMC Public Health, 14:754. 25064309 10.1186/1471-2458-14-754 PMC4124160

[3] Heesterbeek H Anderson RM Andreasen V (2015). Modeling infectious disease Dynamics in the complex landscape of global health. Science, 347(6227):aaa4339. 25766240 10.1126/science.aaa4339 PMC4445966

[4] Osei F B Duker AA Stein A (2012). Bayesian structured additive regression modeling of epidemic data: application to cholera. BMC Med Res Methodol, 12:118. 22866662 10.1186/1471-2288-12-118 PMC3528434

